# Association between consumption of ultra-processed foods and serum C-reactive protein levels: cross-sectional results from the ELSA-Brasil study

**DOI:** 10.1590/1516-3180.2018.0363070219

**Published:** 2019-07-15

**Authors:** Aline Ester da Silva Cruz Lopes, Larissa Fortunato Araújo, Renata Bertazzi Levy, Sandhi Maria Barreto, Luana Giatti

**Affiliations:** I MSc. Dietitian, Postgraduate Program on Health and Nutrition, School of Nutrition, Universidade Federal de Ouro Preto (UFOP), Ouro Preto (MG), Brazil.; II MSc. Dietitian and Adjunct Professor, School of Medicine, Universidade Federal do Ceará (UFC), Fortaleza (CE), and Research Group on Epidemiology of Chronic and Occupational Diseases (GERMINAL), School of Medicine, Universidade Federal de Minas Gerais (UFMG), Belo Horizonte (MG), Brazil.; III PhD. Scientific Researcher VI, Department of Preventive Medicine, Faculdade de Medicina da Universidade de São Paulo (FMUSP), Universidade de São Paulo, São Paulo (SP), Brazil.; IV MD. Physician and Full Professor, Research Group on Epidemiology of Chronic and Occupational Diseases (GERMINAL), School of Medicine & Clinical Hospital, Universidade Federal de Minas Gerais (UFMG), Belo Horizonte (MG), Brazil.; V MD. Physician and Adjunct Professor, Research Group on Epidemiology of Chronic and Occupational Diseases (GERMINAL), School of Medicine & Clinical Hospital, Universidade Federal de Minas Gerais (UFMG), Belo Horizonte (MG), Brazil.

**Keywords:** Diet, C-reactive protein, Inflammation, Obesity, Cross-sectional studies

## Abstract

**BACKGROUND:**

There may be a direct association between consumption of ultra-processed foods and C-reactive protein (CRP) levels, under the assumption that the high glycemic index of these food products could stimulate the entire chronic inflammation cascade, along with an indirect association mediated by obesity. The types of food consumed, including ultra-processed products, strongly influence obesity, and are also associated with higher serum CRP levels.

**OBJECTIVE:**

Our aim was to investigate whether the caloric contribution of ultra-processed foods to diet is associated with CRP levels, independent of body mass index (BMI).

**DESIGN AND SETTING:**

Cross-sectional analysis on the Longitudinal Study of Adult Health (ELSA-Brasil) baseline cohort (2008-2010).

**METHODS:**

Dietary information, obtained through a food frequency questionnaire, was used to estimate the percentage of energy contribution from ultra-processed food to individuals’ total caloric intake. CRP levels were the response variable. Sex-specific associations were estimated using generalized linear models with gamma distribution and log-link function.

**RESULTS:**

Ultra-processed food accounted for 20% of total energy intake. Among men, after adjustments for sociodemographic characteristics, there was no association between ultra-processed food intake and CRP levels. Among women, after adjustment for sociodemographic characteristics, smoking and physical activity, the highest tercile of ultra-processed food intake was associated with mean CRP levels that were 14% higher (95% confidence interval: 1.04-1.24) than those of the lowest tercile. However, after considering BMI, this association lost statistical significance.

**CONCLUSION:**

Our findings suggest that the positive association of ultra-processed food consumption with CRP levels among women seems to be mediated by the presence of adiposity.

## INTRODUCTION

Low-grade chronic inflammation is a mechanism common to many chronic non-communicable diseases that can be measured using biomarkers such as C-reactive protein (CRP).^1^ Health-related behaviors, including diet, may influence the onset and progression of chronic inflammation.^1^ Dietary patterns characterized by high intake of sugars, refined grains, red meat, saturated and trans fats and reduced fiber content have been correlated with higher plasma CRP levels.^2^ However, while some studies have suggested that the association between dietary patterns and CRP levels seems to differ according to sex,^3^^,^^4^^,^^5^ others have not supported this association.^6^^,^^7^

Ultra-processed foods are ready-to-eat industrial formulations that are made entirely or predominantly of substances extracted from foods, food constituents or laboratory-synthesized ingredients based on organic materials. These foods present an unbalanced nutritional composition, lack micronutrients and phytochemicals, contain low levels of fiber and protein and are rich in free sugars, total fats, saturated and trans fats and sodium.^8^

Previous studies have identified positive associations between consumption of ultra-processed foods and obesity,^9^^,^^10^^,^^11^^,^^12^^,^^13^ metabolic syndrome in adolescents,^14^ dyslipidemia in children^15^ and hypertension in adults.^16^ In addition, a recent ecological study that included data from 19 European countries showed a positive association between household availability of ultra-processed foods and the prevalence of obesity among adults.^9^

We did not identify any study investigating the relationship between consumption of ultra-processed foods and inflammatory markers, such as CRP. It is possible that these foods may have a direct association with CRP, under the assumption that the high glycemic index of ultra-processed products could stimulate the entire chronic inflammation cascade,^17^ along with an indirect association mediated by obesity. The types of food consumed, including ultra-processed products, strongly influence obesity ^9^^,^^10^^,^^11^^,^^12^ and are also associated with higher serum CRP levels.^18^

## OBJECTIVE

The aim of the present study was to investigate whether the consumption of ultra-processed foods is associated with CRP levels, regardless of total energy intake, among men and women who were enrolled at the baseline of the Brazilian Longitudinal Study of Adult Health (ELSA-Brasil). In addition, its aim was to determine whether this association is independent from body mass index (BMI).

## METHODS

### Study design, subjects and ethical approval

A cross-sectional analysis was conducted on baseline data (2008-2010) from ELSA-Brasil. This is a multicenter cohort of 15,105 civil servants (aged 35-74 at the time of enrolment) at public universities and research institutions located in six Brazilian states.^19^^,^^20^ The ELSA-Brasil study was approved by the research ethics committees of the six participating institutions (UFMG: ETIC 186/06; HU/USP: 669/06; UFRGS: 194/061; UFES: 041/06; UFBA: 027/06; FIOCRUZ: 343/06), and all participants signed an informed consent form. Details on the study design and cohort profile can be found in other publications.^19^^,^^20^

This analysis excluded subjects with the following characteristics: those with total energy intake (kcal/day) below the 1^st^ and above the 99^th^ percentiles (n = 439); those for whom CRP data was missing (n = 15) and those with CRP values > 99^th^ percentile, which was equivalent to 20.3mg/l (n = 143); those with a history of bariatric surgery (n = 107); those who had undergone changes to their dietary habits within the six months prior to the interview (n = 4,439); and those with the following conditions: diabetes (n=1,322), cardiovascular disease (n = 732) and cancer (n = 686). In the end, our sample consisted of 8,468 subjects.

### Study variables

The response variable of this study was the CRP level (mg/l), measured in blood after 12 hours of fasting by means of high-sensitivity immunochemistry-nephelometry assay (BN II; Siemens). CRPvalues lower than the detection limit (0.175mg/l) were automatically set to half the detection limit (n = 369), i.e. 0.0875mg/l.

The explanatory variable was the percentage energy contribution towards total energy intake that came from ultra-processed foods. Information on dietary consumption was obtained using a semi-quantitative food frequency questionnaire (FFQ), with 114 food items. This FFQ had previously been shown to present satisfactory reliability for all nutrients.^21^

The energy values of foods in the FFQ were estimated based on the following formula: number of servings consumed per occasion x weight/serving size x daily intake frequency x nutritional composition of the food serving. The nutritional composition of the food items was based on the Nutrition Data System for Research (NDSR) from the University of Minnesota, and on the Brazilian Food Composition Table (TACO, acronym in Portuguese) from the Campinas State University (Universidade Estadual de Campinas, UNICAMP).^22^

Foods were classified according to their level of processing using the NOVA classification,^23^ as follows: unprocessed and minimally processed foods; processed food ingredients; processed foods; and ultra-processed foods. The present study considered the percentage of the subjects’ total energy intake that came from ­ultra-processed foods.^24^ To calculate this percentage, calories from this food group were divided by total calories, and then multiplied by 100. Lastly, the percentage energy contribution from ultra-processed foods was categorized into terciles.

The following covariates were included:

1. sociodemographic characteristics: age (used as a categorical variable for descriptive and continuous analyses in the regression models), self-declared race/skin color (white, brown/*pardo*, black, Asian descendant or Brazilian indigenous) and educational attainment (university degree, high school, completed elementary school or incomplete elementary school).
2. health-related behaviors: smoking (never smoked, former smoker or current smoker) and physical activity during leisure time (none, light intensity, moderate intensity or high intensity).^25^
3. BMI, calculated as weight/height², was used to classify subjects according to their nutritional status (eutrophic: BMI<25.0kg/m²;overweight: BMI ≥ 25.0-29.9 kg/m²; and obese: BMI ≥ 30kg/m²)^26^and was used as a continuous variable in regression models.

### Statistical treatment

A descriptive analysis was performed using frequencies or medians (1^st^ and 4^th^ quartiles). The difference in CRP medians based on these variables was evaluated using the Kruskal-Wallis tests, and a trend test was performed for medians, whenever appropriate.

The association between the percentage energy contribution from ultra-processed foods and the CRP levels was estimated using generalized linear models (GLM), with gamma distribution and log-link function. The results were presented as the arithmetic mean ratio (AMR), which expresses the exponential of the regression coefficient (β).

Crude arithmetic mean ratios were firstly estimated (model 0) and then sequential adjustments were made, including the variables of age (model 1), race/skin color and current educational attainment (model 2), smoking and physical activity (model 3) and body mass index (model 4). The models were tested for adequacy. For the linear trend analysis, the terciles of the percentage energy contribution from ultra-processed foods were inserted into the models as a continuous variable. Multiplicative interaction between the percentage energy contribution from ultra-processed foods and sex was investigated by including interaction terms in the adjusted regression models (Model 3 and 4). Evidence of multiplicative interaction between the percentage energy contribution from ultra-processed foods and sex was found (model 4: P-value: tercile 2*female = 0.150; P-value: tercile 3*female = 0.006). Therefore, analyses were presented separately for males and females.

Sensitivity analyses were performed with the following exclusions: 1) subjects with serum CRP > 10mg/l, which may indicate acute inflammation, although these values can also be seen in cases of chronic inflammation;^22^ 2) participants on steroids; and 3) women on contraceptives or hormone replacement therapy. The analyses were done using Stata version 12 (Stata Corporation, College Station, USA).

## RESULTS

Among all the participants, most (52.4%) were women; in both sexes, most were aged 45 to 54 years, self-reported their race/skin color as white and had an undergraduate degree. More than half of all the individuals reported that they had never smoked and were not practicing any physical activity or that it was of light intensity. Approximately 16% of the men and 19% of the women presented BMI≥30 kg/m² (Table 1). Ultra-processed foods contributed almost 23% of the total energy (kcal) intake.

**Table 1.** Descriptive characteristics of the study population according to sex, ELSA-Brasil (2008-2010)

	Male		Female	
	n (4,029)	%	n (4,439)	%
Age* (years)				
35 to 44	1,007	24.9	1,003	22.5
45 to 54	1,670	41.5	1,817	40.9
55 to 64	1,004	24.9	1,247	28.3
65 to 74	348	8.4	372	8.3
Race/skin color*,**				
White	2,154	53.4	2,353	53
Brown (*pardo*)	1,196	29.7	1,173	26.4
Black	498	12.3	716	16.1
Asian	75	1.8	122	2.7
Indigenous	54	1.4	29	0.6
Educational attainment*				
University degree	2,042	51.0	2,483	55.9
High school	1,340	33.0	1,582	35.7
Completed elementary school	329	8.2	219	4.9
Incomplete elementary school	318	7.8	155	3.5
Smoking*				
Never smoked	2,109	52.3	2,778	62.6
Former smoker	1,284	32.2	1,039	23.4
Current smoker	636	15.5	622	14
Physical activity*,**				
None	1,535	38.6	2,225	50.9
Light intensity	1,450	36.4	1,352	30.9
Moderate intensity	636	15.8	576	13.0
High intensity	359	8.9	221	5.0
BMI (kg/m^2^)*,**				
Eutrophic (< 25)	1,594	38.8	2,066	46.5
Overweight (≥ 25.0 to 29.9)	1,767	44.4	1,516	34.1
Obese (≥ 30.0)	665	16.7	855	19.2
CRP level (mg/l)^***^	1.20	0.64-2.5	1.47	0.72-3.39
Energy contribution from ultra-processed foods (kcal)^***^	638.2	427.3-936.6	566.7	384.9-808.8

^*^Data expressed as absolute numbers and percentages. The percentages are rounded, making the total percentage for each characteristic not always equal to 100%. **There may be differences in totals due to loss of information. ^***^Continuous variables. Data expressed as medians and interquartile ranges. BMI = body mass index; CRP = C-reactive protein.

The median CRP level was 1.20mg/l (0.64-2.50) for men and 1.47mg/l (0.72-3.39) for women, and this increased with age only in women (Table 2). For both sexes, individuals with incomplete elementary school, light intensity of physical activity, current smokers and obesity presented higher median CRP levels.

**Table 2.** Median C-reactive protein level and interquartile range (IQR) according to sociodemographic characteristics, behaviors, anthropometric measurements, health conditions and consumption of ultra-processed foods, ELSA-Brasil (2008-2010)

	C-reactive protein level (mg/l)					
	Male (n = 4,029) Median (IQR)		P-value	Female (n = 4,439) Median (IQR)		P-value
Age (years)						
35 to 44	0.98	(0.56-2.14)	0.0001	1.25	(0.58-3.40)	0.0002
45 to 54	1.26	(0.68-2.66)		1.46	(0.74-3.40)	
55 to 64	1.32	(0.70-2.55)		1.55	(0.78-3.37)	
65 to 74	1.24	(0.70-3.11)		1.78	(0.90-3.40)	
Race/skin color						
White	1.18	(0.64-2.37)	0.0003	1.47	(0.71-3.29)	0.0001
Brown	1.29	(0.68-2.76)		1.43	(0.72-3.28)	
Black	1.22	(0.66-2.82)		1.96	(0.83-4.45)	
Asian	0.77	(0.39-1.52)		0.92	(0.43-1.72)	
Indigenous	1.25	(0.68-2.54)		1.08	(0.81-2.26)	
Educational attainment						
University degree	1.05	(0.60-2.14)	0.0001	1.33	(0.66-3.07)	0.0001
High school	1.30	(0.69-2.68)		1.65	(0.77-3.82)	
Completed elementary school	1.33	(0.72-3.03)		1.93	(0.89-4.39)	
Incomplete elementary school	1.80	(0.83-3.63)		2.14	(1.03-5.16)	
Smoking						
Never smoked	1.05	(0.59-2.13)	0.0001	1.41	(0.69-3.28)	0.0003
Former smoker	1.23	(0.68-2.64)		1.55	(0.76-3.34)	
Current smoker	1.81	(0.93-3.65)		1.81	(0.79-3.95)	
Physical activity						
None	1.41	(0.71-3.02)	0.0001	1.77	(0.81-3.98)	0.0001
Light intensity	1.17	(0.65-2.37)		1.40	(0.71-3.03)	
Moderate intensity	1.03	(0.58-2.06)		1.11	(0.59-2.65)	
High intensity	0.99	(0.53-2.14)		1.03	(0.48-1.99)	
BMI (kg/m^2^)						
Eutrophic	0.86	(0.48-1.73)	0.0001	0.92	(0.49-1.90)	0.0001
Overweight	1.30	(0.73-2.64)		1.76	(0.93-3.34)	
Obese	1.97	(1.12-3.98)		3.91	(1.98-6.98)	
Percentage energy contribution from ultra-processed foods						
Tercile 1 (lowest)	1.22	(0.67-2.61)	0.09	1.45	(0.69-3.24)	0.3000
Tercile 2	1.20	(0.64-2.53)		1.47	(0.72-3.42)	
Tercile 3 (highest)	1.18	(0.62-2.32)		1.50	(0.74-3.59)	

Median (IQR): median and interquartile range. The differences between median C-reactive protein levels according to variables were tested using the Kruskal-Wallis test. Differences were considered significant at P-values < 0.05. BMI = body mass index (kg/m^2^).

Among women, after adjustments for sociodemographic characteristics and behaviors, the arithmetic mean CRP level was 14% higher in the highest tercile of the percentage energy contribution from ultra-processed foods (arithmetic mean ratio: 1.14; 95% confidence interval, CI: 1.04-1.24) than in the lowest tercile of consumption. However, when adjusted for BMI (model 4), this association was no longer significant. The ultra-processed food consumption did not remain associated with CRP levels among men (Table 3).

**Table 3.** Univariate and multivariate analyses on the association between the percentage energy contribution towards total energy intake that came from ultra-processed foods (in terciles) and the serum levels of C-reactive protein (CRP), ELSA-Brasil (2008-2010)

Males					
Ultra-processed foods	Model 0	Model 1	Model 2	Model 3	Model 4
	ARM (95% CI)	ARM (95% CI)	ARM (95% CI)	ARM (95% CI)	ARM (95% CI)
Tercile 1 (lowest)	1.00	1.00	1.00	1.00	1.00
Tercile 2	0.95 (0.87-1.03)	0,97 (0.89-1.05)	0.99 (0.91-1.08)	0.98 (0.90-1.07)	0.98 (0.90-1.07)
Tercile 3 (highest)	0.85 (0.77-0.92)***^&^	0,88 (0.81-0.96)**^&^	0.93 (0.85-1.02)	0.93 (0.84-1.02)	0.93 (0.84-1.02)
Females					
Ultra-processed foods	Model 0	Model 1	Model 2	Model 3	Model 4
	ARM (95% CI)	ARM (95% CI)	ARM (95% CI)	ARM (95% CI)	ARM (95% CI)
Tercile 1 (lowest)	1.00	1.00	1.00	1.00	1.00
Tercile 2	1.04 (0.95-1.13)	1.04 (0.96-1.14)	1.07 (0.98-1.17)	1.06 (0.98-1.16)	1.01 (0.93-1.10)
Tercile 3 (highest)	1.08 (1.00-1.17)*^&^	1.09 (1.01-1.19)*^&^	1.14 (1.04-1.24)**^&^	1.14 (1.04-1.24)**^&^	1.00 (0.92-1.08)

Ultra-processed foods: percentage energy contribution towards total energy intake that came from ultra-processed foods, in terciles.

ARM (95% CI) = arithmetic mean ratio and 95% confidence interval estimated using a generalized linear model.

Model 0 = crude analysis; model 1 = adjusted for age (continuous); model 2 = model 1 + race/skin color and educational attainment; model 3 = model 2 + smoking and physical activity; model 4 = model 3 + body mass index (kg/m^2^).

*P-value < 0.05; **P-value < 0.01; ***P-value < 0.001; ^&^P-value < 0.05 for linear trend in the association between the percentage energy from ultra-processed foods (in terciles) and CRP.

Sensitivity analyses excluding individuals with serum CRP>10mg/l and those on steroids did not change the results observed among either men or women; nor did analyses excluding women on contraceptives or hormone replacement. When the same analyses were conducted including those who had changed their eating habits within the last six months, the results did not change.

## DISCUSSION

Our results showed that there was a direct association between consumption of ultra-processed foods and CRP levels after adjusting for sociodemographic characteristics and health-related behaviors among women. However, this association disappeared after adjustment for BMI. Among men, there was an inverse association between consumption of ultra-processed foods and CRP levels in the crude analysis and after age adjustment. This association ceased to be significant after adjusting for sociodemographic factors.

This direct association between higher consumption of ultra-processed foods and CRP levels, independent of the total energy intake, corroborates previous studies that pointed out a relationship between unhealthy dietary patterns and higher CRP levels.^28^^,^^40^ Nonetheless, the association observed was specific for women and, after adjusting for BMI, it lost statistical significance. This suggests that the relationship between ultra-processed foods and higher serum CRP levels was completely mediated by adiposity. It is important to highlight that in the present study, consumption of ultra-processed foods was corrected according to the total energy of the diet, i.e. the association found was independent of the total energy intake.

The difference regarding sex that we observed in this analysis is intriguing. Although previous studies have suggested that a sex-specific relationship between diet and CRP level exists, the results have not been consistent. In a Japanese study, the bread pattern (high in bread, margarine and coffee; low in rice and miso soup) and the dessert pattern (high in Western/Japanese confections and fruit) showed inverse associations with CRP levels among men, while the Western pattern (high in meat, eggs, mayonnaise and deep or stir-fried foods) showed a positive association with CRP levels among women.^3^ A multi-city cohort in South America showed that higher intakes of fruits, vegetables, fish, seafood, whole cereal and low-fat dairy products were associated with reduced CRP levels only in men.^4^ On the other hand, a lack of association between ­ultra-processed foods and overweight and obesity among men has already been reported,^10^ as has a stronger association between higher consumption of ultra-processed food and obesity among women.^29^

The positive association between consumption of ultra-processed foods and CRP that has been seen among women may be partly explained by the greater accumulation of body fat in women,^31^ since BMI has been more strongly associated with CRP levels among females, whereas central adiposity seems to be more strongly related to CRP levels among men.^32^^,^^33^ However, other studies have shown higher CRP levels in women than in men, irrespective of potential confounding factors such as race, BMI and estrogen use.^31^^,^^34^ Wealso included waist circumference in the models, but the result did not change (results not shown).Moreover, a meta-analysis on longitudinal studies indicated that diets with high glycemic load and glycemic index, which are both characteristics of ultra-processed foods, were associated with metabolic changes among women, while the results for men were inconsistent.^35^

As in the present analysis, some studies have also shown that the association between the Western dietary pattern, consisting mostly of ultra-processed foods, and increased CRP levels, is attenuated after adjustment for BMI or waist circumference.^36^^,^^37^ A direct relationship between consumption of ultra-processed foods and obesity has already been described.^9^^,^^10^^,^^11^ Ultra-processed food consumption has also been correlated with increased BMI and waist circumference after simultaneous adjustment for these variables and other confounding factors in ELSA-Brasil.^13^ It is well established that adipose tissue produces cytokines that induces CRP production.^38^ Thus, the association between consumption of ultra-processed foods and the inflammatory response is expected to be largely dependent on adiposity.

However, it would be plausible to assume that part of this association is independent from adiposity, since some nutritional characteristics of ultra-processed foods, such as high energy density, high glycemic load and high content of saturated and trans fats,^39^may stimulate inflammatory markers^40^ through promoting oxidative stress. This induces production of free radicals^19^ or suppresses the antioxidant capacity of foods, and leads to hypersecretion of ­pro-inflammatory cytokines.^41^ As mentioned earlier, the total energy intake was corrected through creation of the variable of percentage energy contribution from ultra-processed foods and, therefore, this factor did not influence the results. Our findings did not support this potential relationship between the contribution of ultra-processed foods towards total energy intake and the levels of a chronic systemic inflammation indicator, which in this case was CRP.

One point to be considered in the results from this study is the percentage energy contribution from ultra-processed foods. The NOVA classification proposes an indicator to measure the nutritional quality of a diet by grouping several foods into four groups, which are investigated in terms of energy consumption. It may be that other aspects of the diet that have pro-inflammatory and anti-inflammatory potential could add greater specificity to the diet and contribute towards better understanding of its adverse effects. Moreover, adding other biomarkers or a combination of inflammatory markers may contribute towards investigation of the association between ultra-processed foods and inflammation.

The strengths of this study include its sample size, the quality of data collection, the possibility of enabling adjustment for a large number of potential confounders and the use of a validated food frequency questionnaire (FFQ). Among its limitations, we emphasize that we used an FFQ that can overestimate consumption, especially given that it contained more than 100 food items.^42^ It should also be noted that this FFQ was not designed to assess food consumption based on the level of processing, which may have led to erroneous classification of food items. It is also worth noting that classification of food items based on the level of processing is still a recent concept, and it may be subject to updates and future changes. Another limitation of this study was its cross-sectional design, which made it impossible to establish a temporal relationship between consumption of ultra-processed foods and CRP levels.

## CONCLUSION

This study provides a contribution to the recent literature focusing on investigation of the relationship between consumption of ultra-processed foods and metabolic changes, especially those relating to chronic non-communicable diseases. Our findings suggest that there is a positive relationship between ultra-processed foods and CRP levels among women, irrespective of total caloric intake; however, this association appears to be totally dependent on adiposity. Thus, our results indicate that cutting back on ultra-processed foods can decrease chronic low-grade inflammation, even if through reducing obesity. This reinforces the importance of public policies aimed towards restricting the availability of ultra-processed foods.
